# Effects of Flywheel Training on Strength-Related Variables: a Meta-analysis

**DOI:** 10.1186/s40798-018-0169-5

**Published:** 2018-12-13

**Authors:** Henrik Petré, Fredrik Wernstål, C. Mikael Mattsson

**Affiliations:** 1 Åstrand Laboratory of Work Physiology, The Swedish School of Sport and Health Sciences, Stockholm, Sweden; 2 Department of Physiology and Pharmacology, Karolinska Institutet, SE-171 77 Stockholm, Sweden; 3 Silicon Valley Exercise Analytics, Menlo Park, CA USA

**Keywords:** Muscle hypertrophy, Maximum strength, Power, Vertical movement, Horizontal movement

## Abstract

**Background:**

Strength and power development are abilities important for athletic performance in many sports. Generally, resistance training based on gravity is used to improve these qualities. Flywheel training instead utilizes kinetic energy transferred to a flywheel. This allows for eccentric overload and variable resistance throughout the movement.

The aim of this review was to identify the effects of flywheel training on multiple strength-related variables affecting athletic performance. The meta-analysis investigates the effects on (1) muscle growth (cross-sectional area (CSA) and volume/mass), (2) maximum dynamic strength, (3) development of power, (4) development of horizontal movement, and (5) development of vertical movement.

**Methods:**

The meta-analysis includes 20 experimental studies that met the inclusion criteria. The quality of included studies was ranked according to the PEDro scale. Possible bias was identified in Funnel plot analyses. To enable the compilation of all results analyses, the random effect model was carried out using the software Review Manager Version 5.3 and presented with Forest plots.

**Results:**

Flywheel training for a period of 4–24 weeks shows statistically significant increases in all strength aspects. Effect sizes were for hypertrophy, CSA 0.59; volume/mass 0.59; maximum strength 1.33; power 1.19; horizontal 1.01 and vertical movement 0.85. The evidence is particularly strong for beneficial effects from flywheel training in the development of maximal strength and power in trained younger individuals, and utilization of this training modality in shorter more intensive blocks.

**Conclusions:**

Flywheel training is an effective method for improving several aspects of strength and power with importance for sports performance.

**Electronic supplementary material:**

The online version of this article (10.1186/s40798-018-0169-5) contains supplementary material, which is available to authorized users.

## Key Points

- Flywheel training is a strength training modality that offers the possibility of performing exercises with eccentric overload and variable resistance as compared to conventional gravity-based resistance training.
- Flywheel training seems to be a viable alternative to regular resistance exercise with comparable positive strength and hypertrophic adaptations in untrained, moderately trained, and well-trained individuals, with, surprisingly, greater strength improvements in the well-trained group, and among younger individuals.

## Background

Strength and strength-related variables are important components for performance in many sports. The ability of the neuromuscular system to produce force against an external load is a definition of strength. The high requirement for sport-specific training in many complex sports allows for less time and focus for improvement of other more general but important qualities, like strength. Therefore, strategies employed to increase the efficiency of strength development are of great importance for developing resistance to injury and optimization of athletic performance.

**Fig. 1:**
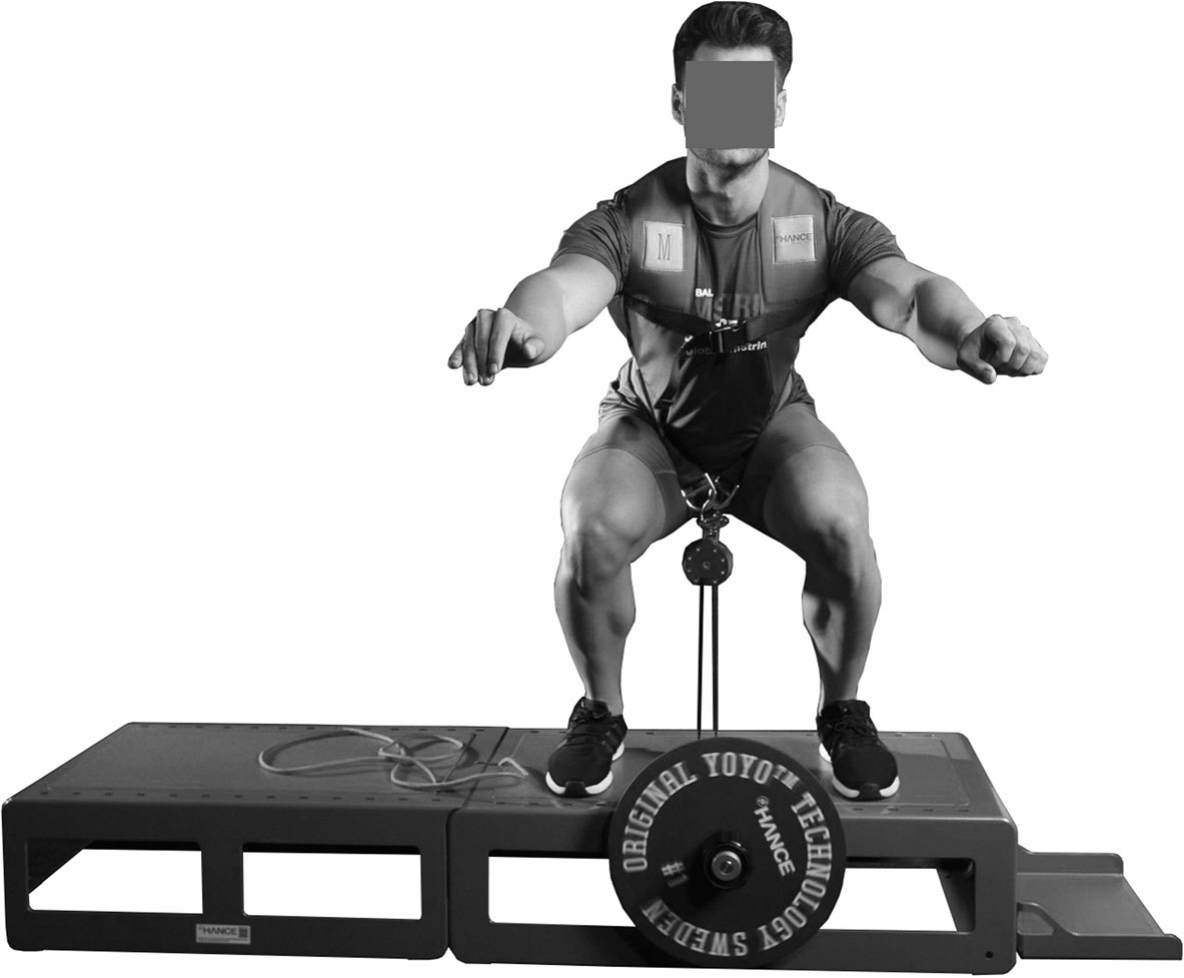
A typical flywheel machine. Pictured is YoYo™ Ultimate Squat (model #215) with Hooper’s Box. Courtesy by nHANCE™ driven by YoYo™ Technology—Copyright© 2018. All rights reserved

Many different methods to improve strength have been suggested throughout the years, including the use of free weights, weight stacks, resistance bands, and machines using liquid or air pressure as resistance [1]. A training method to develop strength that has increased in popularity during the past decades is flywheel training. Such devices consist of one or more flywheels connected to a rotating shaft (Fig. 1). By pulling a band wound on a shaft, the flywheel starts rotating. The concentric muscle activation thus transfers kinetic energy to the flywheel. When the band is pulled to its maximum length, the flywheel continues to spin and winds the band on the shaft again requiring eccentric muscle action to slow the kinetic energy of the flywheel. The more inertia (kg m^2^), by using larger or additional flywheels, the more force is required to increase the speed of the flywheel [2]. Lower body eccentric training with flywheel machines is mainly performed with a harness. Utilizing a harness can reduce injury risk by distributing the center of gravity throughout the movement, and thereby decreasing the length of the movement arm. In this way, the stress and strain on the lower back in a squat can be limited. The biomechanical benefits of a harness in flywheel training decrease the technical skills necessary for proper movement execution.

The aims of strength training are development of muscular hypertrophy, maximal strength (force development), and/or power. An increase in hypertrophy improves the possibility of developing force. There seems to be a proportional relationship between hypertrophy and force. Power development is a product of force multiplied by the velocity of the motion. Hence, there is a correlation between force and power which is in line with Newton’s second law of motion which specifies that a greater force generates increased acceleration if the mass of the object is constant. [3–6].

Strength and power correlate well with performance in multiple sports requiring motion in the vertical and horizontal plane [1, 7, 8]. Motion in the horizontal plane (by acceleration) and “flying” sprints are mainly dynamic repetitive movements that, on top of the initial concentric contraction creating acceleration, stress the ability to eccentrically slow down and stop the motion, whereupon starting a new acceleration phase [9]. Eccentric and concentric strength is thus of importance. A greater ability of rapidly slowing the eccentric motion contributes to an increased amount of elastic energy build up in the tissues and therefore an increased effect of the stretch-shortening can be utilized. This contributes to an increase in force in the concentric phase of the motion [10, 11].

Traditional gravitational-dependent resistance training with free weights or weight stacks involves muscle action against a constant external load [12]. The load in the concentric phase of the movement in traditional resistance training requires adaptation to the angle in the range of motion where the external moment arm is the longest. The length of the external moment arm varies with joint angle and the ability of the muscle to develop force is affected by the length of the muscle and the internal moment arm (the moment arm of the muscle), resulting in incomplete activation of muscle motor units in traditional resistance training [12, 13]. In flywheel training, on the other hand, the resistance is created by an inner inertia in one or more flywheels. Flywheel training thereby allows for maximal resistance throughout the whole range of motion and in every single repetition in a set, irrespective of the internal and external moment arm [2, 12, 14–17]. By adjusting the number of flywheels, it is possible to attune the speed of the movement and thereby manipulate the training to achieve the desired training adaptations.

Flywheel training allows for, not only maximal muscle activation in the concentric phase but also for short periods of increased resistance in the eccentric phase compared to the concentric phase, also known as overload [2, 12]. For example, overload is created by resisting the eccentric force later in the eccentric range of motion. An overload in the later stage of the eccentric phase of the motion is possible even with conventional load alternatives, like dumbbell and barbell exercises. However, the velocity of the object (e.g., dumbbell or barbell) in the eccentric phase makes the object significantly more difficult to handle, possibly increasing injury risk. In flywheel training, the kinetic energy is almost constant and independent of duration until eccentric muscle action to slow down the eccentric phase of the lift. This means that overload can be achieved in any part of the eccentric phase and in a more controlled manner. Furthermore, by increasing the force applied in the concentric phase, e.g., by the help of a trainer or by utilizing different muscles, one can transfer more kinetic energy to the flywheel, thereby creating more overload.

Unlike strength training using free weights, the load on the targeted muscles will not be affected if other muscles enter and assist in the concentric phase because the force is applied against an intrinsic inertia when using flywheel-based hardware.

Any type of training with eccentric overload is effective in promoting muscular hypertrophy [18] and maximal strength [3, 19–21]. A recently published meta-analysis concluded flywheel training with overload was superior for muscular hypertrophy, maximal strength, and power compared to traditional strength training [22]. In addition, a study that investigated the effects of eccentric overload training compared to isoinertial exercise showed greater increases in maximal force production but no change in cross-sectional area (CSA) in the eccentric overload group [23]. In support of this, a recent review concludes the use of eccentric overload, not limited by concentric strength, could be superior to traditional resistance training with regards to variables associated with strength, power and speed performance [24]. Even though superiority is questionable, flywheel training was shown in several studies to be effective in developing muscular hypertrophy, maximal strength, and power [6, 12, 15, 16, 25–27]. The results of functional tests in the vertical and horizontal plane, e.g., vertical jump, sprints, and directional changes, showed positive results in both acute and long-term studies [10, 28, 29].

Although flywheel training had positive effects on strength, few systematic reviews or meta-analyses were done with the aim of compiling its effects on strength and power outcomes. A limitation with the existing studies is the use of notably different protocols and executions. For example, differences in muscle groups trained, sets and repetitions performed, measuring tools and inertia used, the age of the participants, and training experience are some variables that vary widely among studies.

Meta-analyses were published on the effects of flywheel training on the improvement of muscle strength compared to conventional gravity-dependent resistance training [30], eccentric overload and effects on muscle size and functional capacities in athletes and healthy subjects [22], or effects of chronic flywheel training on muscle volume and force [31]. However, our analysis includes more studies than previous meta-analyses, more recently published data, as well as comparative analyses on the effects of flywheel training on well-trained individuals and novices. Therefore, the primary aim of this meta-analysis was to identify the effect of flywheel training on strength-related variables affecting athletic performance by examining and compiling relevant studies.

The objectives of this analysis were to investigate the effect of flywheel training on muscle hypertrophy (CSA and volume/mass), development of maximal dynamic strength, development of power, displacement in the horizontal plane, and displacement in the vertical plane.

## Methods

This meta-analysis was conducted in accordance with Preferred Reporting Items for Systematic Reviews and Meta-analyses (PRISMA) statement guidelines [32, 33].

### Search Strategy

**Fig. 2:**
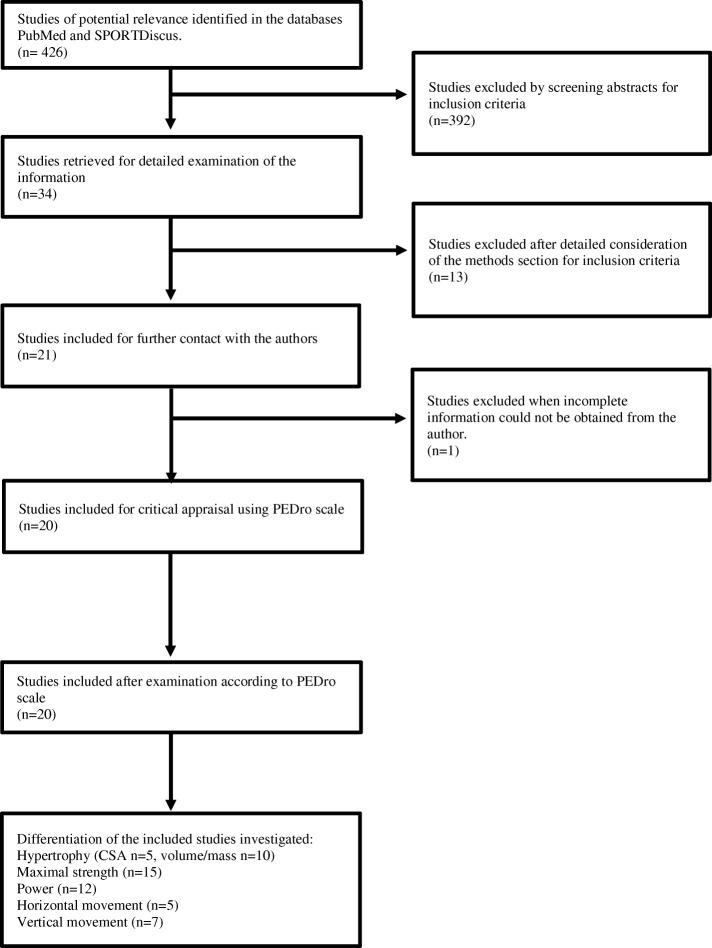
Flow chart of study selection for inclusion in the meta-analysis. *n* number of studies, *CSA* cross-sectional area

Original articles published in English before August 2018 were located using the databases PubMed and SPORTDiscus. The search terms used to identify potential articles for inclusion were “flywheel training,” “inertia training,” “flywheel inertia,” “flywheel resistance training,” “flywheel resistance exercise,” “training eccentric overload,” “flywheel muscle exercise,” “isoload,” and “isoinertial.” These search terms were used due to their association with flywheel training. The authors of the 20 articles meeting the inclusion criteria for final analysis (Fig. 2) were contacted if data relevant for this meta-analysis were lacking.

### Study Selection

#### Type of Studies

Only original articles with an experimental design using training interventions lasting between 4 and 24 weeks were included in this meta-analysis, thus, no acute studies were included.

#### Study Participant Characteristics

Only studies in healthy men and women, without age-restriction, were included in this meta-analysis. In the present review, the participants were divided between *untrained*, *moderately trained*, and *well*-*trained*. Classified as *untrained* were individuals with no or minimal experience of resistance training and individuals without the participation of programmed physical exercise during the last six months. *Moderately trained* individuals were those who reported as recreationally active and moderately active. If no distinction was apparent between untrained and moderately trained in the studies, the individuals were classified as moderately trained. Classified as *well*-*trained* were sport-participating individuals, elite athletes, and individuals with self-reported high activity level.

#### Type of Intervention

Only studies measuring flywheel training and its effects on muscular hypertrophy, maximal dynamic strength, power, and displacement in the horizontal and vertical plane were included in this meta-analysis. Four studies included a non-training passive control group [6, 15, 26, 34]. Three studies performed flywheel training in-season [30, 35, 36], two of which performed additional training for the experimental group in addition to the standard training performed by both groups [29, 37]. One of the studies included a control group performing conventional resistance training, including free weight strength training [14]. Measurements of acceleration during 10 and 20 m and “flying” sprints was included in horizontal displacement. In this paper, countermovement jump, vertical jump, and drop jump were included in the category displacement in the vertical plane.

#### Measuring Instruments

Studies measuring hypertrophy (CSA or volume/mass) using magnetic resonance imaging (MRI), dual-energy X-ray absorptiometry (DXA), or bioelectrical impedance analysis (BIA) were included in this meta-analysis. Studies using power measurements in the form of isokinetic devices, linear encoders, or any form of measuring devices connected to the flywheel apparatus when training were approved for inclusion. Maximal strength measured with free weights (1RM or 3RM) and hydraulically driven devices with isokinetic dynamometers were included in this meta-analysis. Only studies measuring horizontal displacement using photocells or timing gates were included. Force plates were considered the gold standard for measuring vertical jump height [35]. Vertical displacement measured using an optical sensor, contact plate, or with a measuring stick with centimeter scale was included. Optical sensors have been validated against force plates for squat jump and countermovement jump and have shown strong concurrent validity and excellent test-retest reliability [38]. Measuring changes in jump height with jump-mat has also proven valid [39].

#### Presented Results

Only studies presenting raw data in the form of absolute values, or for which absolute values were obtained from the authors upon request, were included in the final analysis.

#### Exclusion Criteria

Studies were excluded if the study participants were in a state of energy deficit during the study period, in other catabolic states like for example bed rest, under intake of any medical supplement, or if the study used engine-driven flywheel machines, or a flywheel machine without a straight shaft. In cases where complete information, i.e., absolute values, of the variables investigated in present meta-analysis were not obtained after contact with the authors, the studies were excluded.

#### Selection Process

The selection process is outlined in Fig. 2. The initial search identified 426 studies of potential relevance. However, after applying and screening for the inclusion and exclusion criteria specified above, 20 studies met the inclusion criteria and were thus included in the meta-analysis [2, 6, 12, 15, 16, 26, 27, 29, 34, 36, 37, 40–48].

### Quality Assessment

To increase the quality of the meta-analysis, analyses, and conclusion, quality control was utilized [49]. The methodological quality of all included studies was assessed using the Physiotherapy Evidence Database (PEDro) scale. Only studies exceeding three points on the PEDro scale, meaning medium-level evidence, were included in this meta-analysis (Pedro.org.au). The quality assessment was performed, in concordance with the recommendations, by two independent researchers [49].

A funnel plot measures every individual study’s effect based on the size of the study in relation to the difference between pre and post-test. If a funnel plot shows a symmetrical shape centered around the area of mean effect of the studies, the identification and selection process are considered to be devoid of bias [49]. Funnel plots are presented as Additional file 1: Figures S1–S6.

### Statistics and Data Analysis

**Fig. 3:**
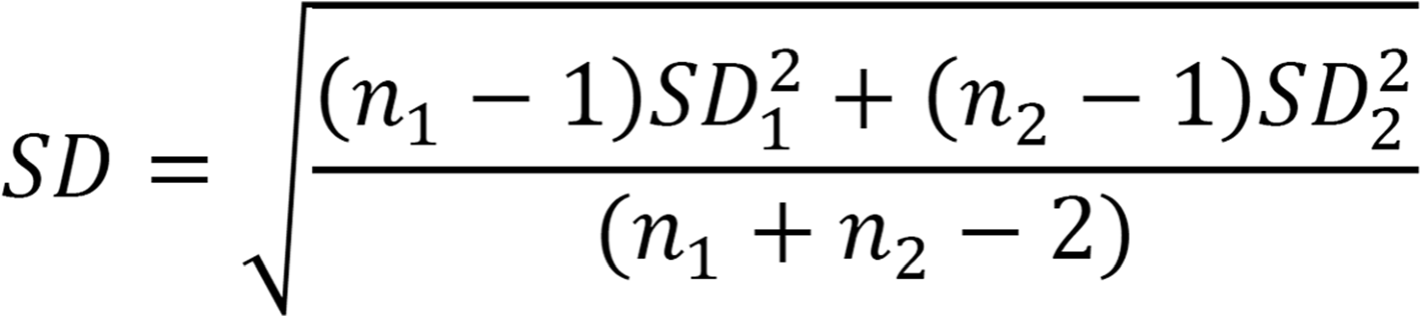
The equation used for the calculation of pooled standard deviations. *SD* standard deviation, *n* number of study participants

To compare and quantify the results of the included studies, all gathered data were analyzed using the random effect model and presented in Forest plots. The obtained values from the included studies were analyzed using the program Review Manager (RevMan. Version 5.3. Copenhagen: The Nordic Cochrane Centre, The Cochrane Collaboration, 2014). Data were gathered from each study, and required a mean value and ± standard deviation (SD) from pre- and post-test to be included. Data were then published as the differences in means in our analyses. All studies could include one value for every strength-related variable. Studies containing several values for the same variable were added and the SD was pooled according to the equation presented in Fig. 3.

The effect sizes are presented in Forest plots with 95% confidence interval (CI). The effect sizes are defined as negligible (< 0.2), small (0.2–0.49), moderate (0.5–0.79), large (0.8–1.19), very large (1.2–1.9), or extremely large (> 2.0) in accordance to Cohen [50] and Sawilowsky [51]. Significance levels were set to *p* < 0.05. The changes between pre- and post-test are also presented with a percentage, which is weighed in relation to the number of participants.

## Results

The 20 selected studies and subject characteristics are presented in Table 1. One study presenting data on vertical and horizontal displacement was excluded due to lack of presented information [10]. The size of the intervention groups in the included studies ranged from 7 to 37 and ages 17–69 years. Fourteen studies only included male participants, no study used solely female participants, and 7 studies included participants from both sexes. Regarding exercise selection, there was a large variation (4 studies used squat, 2 used leg press, 11 used knee extension, 2 used leg curl, 2 used lunges, 1 used shoulder adduction and abduction, and 1 used elbow flexion and extension).

**Table 1:** Characteristic of the included studies and calculated training effects for strength and results on functional tests

Study	Characteristics of participants		Intervention					Effects of flywheel training
	*n*/ sex/age (year)	Fitness level	Exercise	Reps or s x set	Rest between set (s)	Period (w)	Session/w	Results (%)
Askling et al. 2003 [40]	15/M/26 ± 4	Well-trained	Leg curl	8 × 4	60	10	1–2	Horizontal movement (time): − 2.4 Maximal strength: + 17.1
Bruseghini et al. 2015 [36]	12/M/68 ± 4	Moderately trained	Knee extensor	7 × 4	180	8	3	Hypertrophy (CSA): + 4.2 Hypertrophy (Volume/mass): + 4.9 Maximal strength: + 6.5
Caruso et al. 2005 [41]	10/M + F/59 ± 2	Untrained	Leg press	8 × 4	90	10	3	Maximal strength: + 2.5 Hypertrophy (Volume/mass): + 3.9
De Hoyo et al. 2015 [29]	18/M/18 ± 1	Well-trained	Squat and leg curl	6 × 3–6	180	10	1–2	Horizontal movement: − 1.5 Vertical movement: + 7.3
Fernandez-Gonzalo et al. 2014 [27]	32/M + F/24 ± 1	Moderately trained	Leg press	7 × 4	180	6	2–3	Hypertrophy (volume/mass): + 5.0 Maximal strength: + 22.4 Power: + 5.3 Vertical movement: + 5.8
Gual et al. 2016 [37]	27/M + F/23 ± 4	Well-trained	Squat	8 × 4	120	24	1	Power: + 59.2 Vertical movement: + 3.4
Lundberg et al. 2013 [42]	10/M/25 ± 4	Moderately trained	Knee extensor	7 × 4	120	5	2–3	Hypertrophy (CSA): + 10.8 Hypertrophy (Volume/mass): + 10.7 Maximal strength: + 28.5 Power: + 26.1
Lundberg et al. 2014 [43]	10/M/26 ± 5	Moderately trained	Knee extensor	7 × 4	120	5	2–3	Hypertrophy (CSA): + 4.1 Hypertrophy (Volume/mass): + 4.2 Maximal strength: + 10.8 Power: + 17.1
Maroto-Izquierdo et al. 2017 [44]	15/M/20 ± 1	Well-trained	Knee extensor	7 × 4	180	6	3	Maximal strength: + 11.8 Power: + 15.3 Horizontal movement (time): − 10.8 Vertical movement: + 9.8
Naczk et al. 2014 [6]	33/M/21 ± 1	Well-trained	Shoulder add och abd	20 s × 3	120	4	3	Maximal strength: + 13.5 Power: + 14.8
Naczk et al. 2016 [26]	37/M/21 ± 1	Well-trained	Knee extensor	15 s × 3	120	5	3	Hypertrophy (Volume/mass): + 11.0 Maximal strength: + 25.7 Power: + 32.6 Vertical movement: + 10.8
Naczk et al. 2016 [34]	20/M/22 ± 1	Well-trained	Elbow flexor and extensor	15 s × 3	120	5	3	Hypertrophy (Volume/mass): + 13.7 Maximal strength: + 14.4 Power: + 17.8
Norrbrand et al. 2008 [2]	7/M/39 ± 9	Untrained	Knee extensor	7 × 4	120	5	2–3	Hypertrophy (Volume/mass): + 6.2 Maximal strength: + 7.6 Power: + 10.5
Norrrbrand et al. 2010 [12]	9/M/39 ± 5	Untrained	Knee extensor	7 × 4	120	5	2–3	Maximal strength: + 8.2
Núñez et al. 2018 [45]	27/M/23 ± 3	Well-trained	Squat and lunges	7 × 4	180	6	2	Hypertrophy (Volume/mass): + 6.1 Power: + 30.0 Horizontal movement (time): + 0.03 Vertical movement: + 5.0
Onambele et al. 2008 [46]	27/M/23 ± 3	Untrained	Knee extensor	8–12 × 1–4	300	12	3	Power: + 27.8
Owerkowicz et al. 2016 [47]	17/M + F/22 ± 1	Untrained	Knee extensor	7 × 4	300	5	2	Hypertrophy (CSA): + 9.9 Maximal strength: + 16.5
Sabido et al. 2017 [48]	11/M/24 ± 4*	Well-trained	Squat and lunges	8 × 4 and 8 × 2	120	7	1	Maximal strength: + 14.2 Power: + 38.2 Horizontal movement (time): −2.5 Vertical movement: + 6.0
Seynnes et al. 2007 [15]	7/M + F/20 ± 2	Moderately trained	Knee extensor	7 × 4	120	5	3	Hypertrophy (CSA): + 6.9 Maximal strength: + 38.7
Tesch et al. 2004 [16]	10/M + F/39 ± 8	Moderately trained	Knee extensor	7 × 4	120	5	2–3	Hypertrophy (Volume/mass): + 6.1

*M* man, *F* female, *Add* adduction, *abd* abduction, *s* seconds, *w* weeks, * combined age for both training and control groups

Nine of the analyzed studies included well-trained individuals, six of the studies included moderately trained individuals, and five studies included untrained individuals. The degree of inertia in the included studies varied between 0.07 and 0.145 kg m^2^. The rest times between sets varied between 1 and 5 min (1 study had 1 min, 1 study had 1.5 min, 11 studies had 2 min, 5 studies had 3 min, and 2 studies had 5 min). In four of the studies [37, 44, 52], the intervention period coincided with the competition season and the other 16 studies were performed during pre-season or when there was no other physical activity at the time of the study period.

### Strength

#### Hypertrophy

**Fig. 4:**
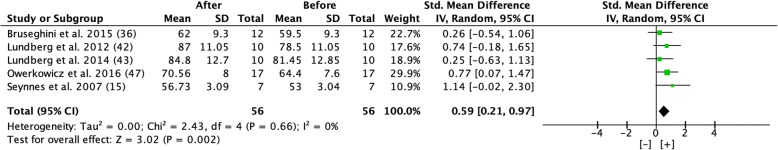
Forest plot showing effect size comparing pre and post-test muscle cross-sectional area during a period of 5 to 8 weeks of flywheel training [15, 36, 42, 43, 47]. [+] indicates positive effect of flywheel training. *SD* standard deviation, *Std* standardized, *IV* inverse variance, *CI* confidence interval

**Fig. 5:**
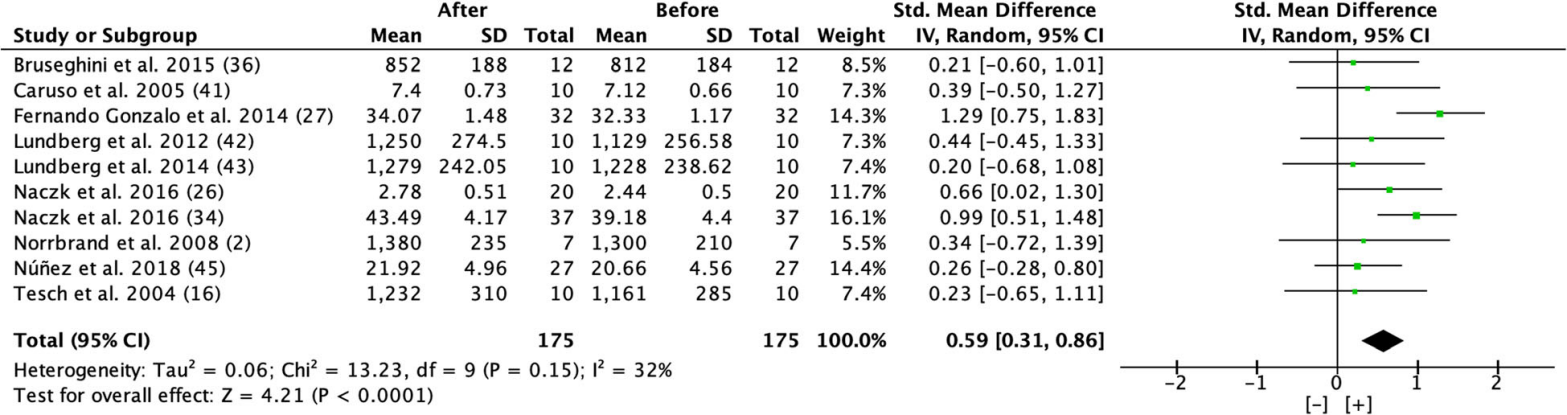
Forest plot showing effect size comparing pre and post-test muscle volume/mass during a period of 5 to 8 weeks of flywheel training [2, 16, 26, 27, 34, 36, 41–43, 45]. [+] indicates positive effect of flywheel training. *SD* standard deviation, *Std* standardized, *IV* inverse variance, *CI* confidence interval

Overall, there was a significant increase in hypertrophy of 7.4% in muscle CSA and 7.8% in muscle volume/mass with moderate effect sizes after 5–8 weeks of flywheel training 2–3 times per week (Figs. 4 and 5).

#### Maximal Strength

**Fig. 6:**
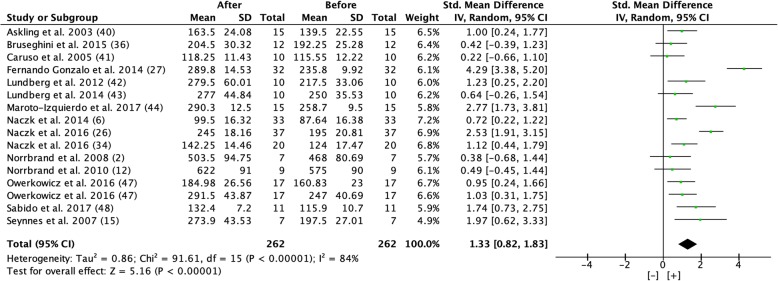
Forest plot showing effect size comparing pre and post-test maximal strength during a period of 4 to 10 weeks of flywheel training [2, 6, 12, 15, 26, 27, 34, 36, 40–44, 47, 48]. [+] indicates positive effect of flywheel training. *SD* standard deviation, *Std* standardized, *IV* inverse variance, *CI* confidence interval

Overall, a significant increase of 17.3% in maximal strength was seen with a very large effect size from pre- to post-test (Fig. 6). The post-test was completed after 4–10 weeks of flywheel training 1–3 times per week.

#### Power

**Fig. 7:**
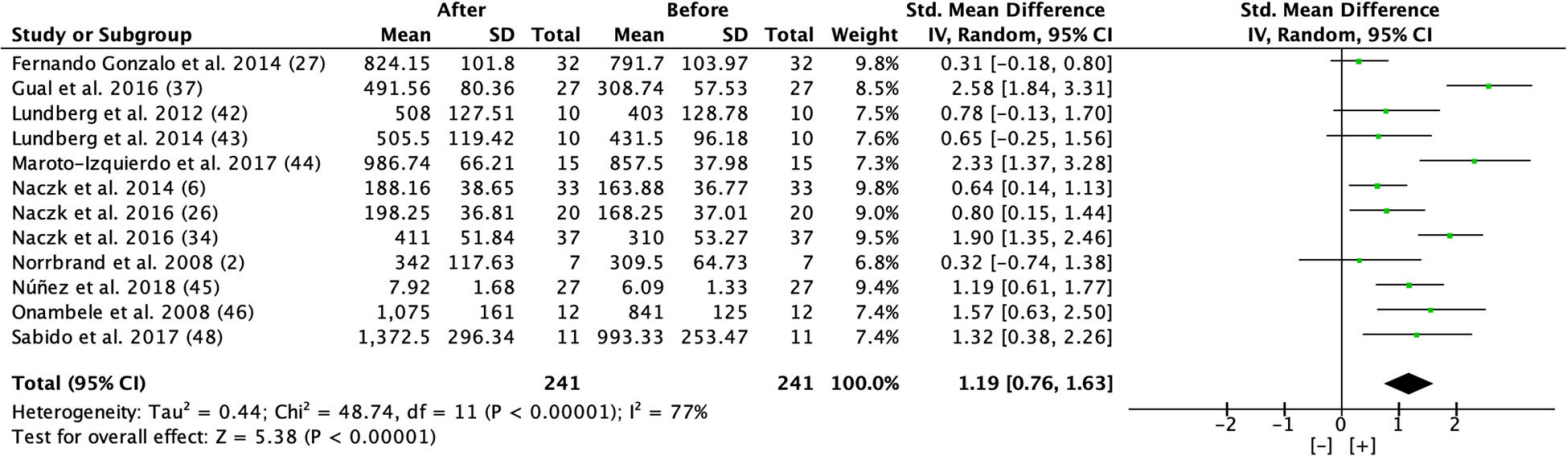
Forest plot showing effect size comparing pre and post-test power during a period of 4 to 24 weeks of flywheel training [2, 6, 26, 27, 34, 37, 42–46, 48]. [+] indicates positive effect of flywheel training. *SD* standard deviation, *Std* standardized, *IV* inverse variance, *CI* confidence interval

Overall, a significant increase of 25.2% in power was seen with a large effect size from pre- to post-test (Fig. 7). The post-test was completed after 4–24 weeks of flywheel training 1–3 times per week.

### Functional Tests

#### Horizontal Displacement

**Fig. 8:**
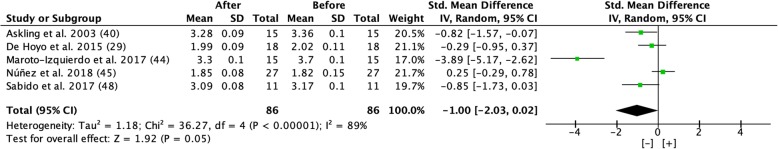
Forest plot showing effect size comparing pre and post-test horizontal displacement during a period of 6 to 10 weeks of flywheel training [29, 40, 44, 45, 48]. [−] indicates positive effect of flywheel training, i.e. decreased times. *SD* standard deviation, *Std* standardized, *IV* inverse variance, *CI* confidence interval

Overall, a significant improvement of 2.4% in horizontal displacement (i.e., decreased times) was seen with a large effect size from pre- to post-test (Fig. 8). The post-test was completed after 6–10 weeks of flywheel training 1–3 times per week.

#### Vertical Displacement

**Fig. 9:**
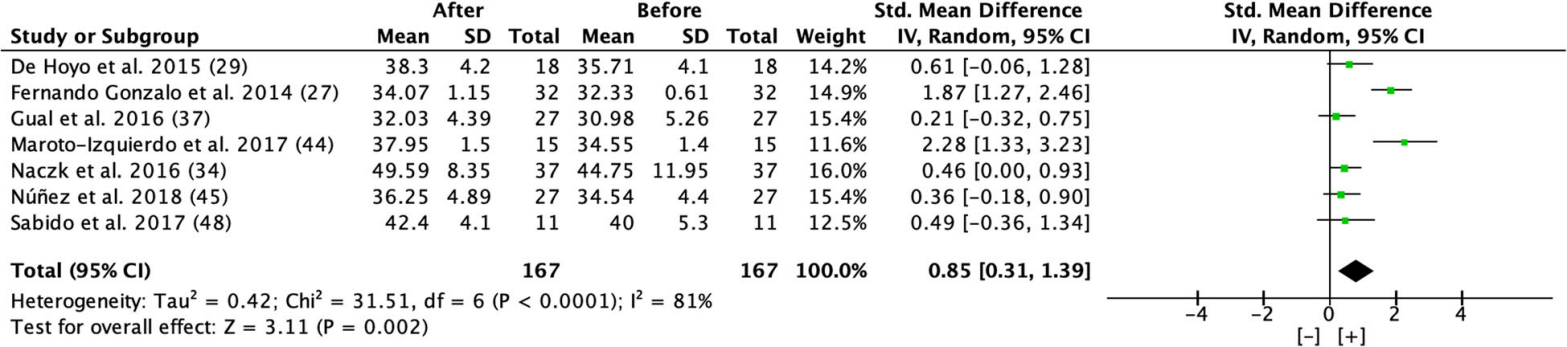
Forest plot showing effect size comparing pre and post-test vertical displacement during a period of 5 to 24 weeks of flywheel training [27, 29, 34, 37, 44, 45, 48]. [+] indicates positive effect of flywheel training. *SD* standard deviation, *Std* standardized, *IV* inverse variance, *CI* confidence interval

Overall, a significant increase of 6.8% in vertical displacement was seen with a large effect size from test pre- to post-test (Fig. 9). The post-test was completed after 5–24 weeks of flywheel training 1–3 times per week.

## Discussion

In total, 20 studies met the inclusion criteria and measured one or more of the effects investigated. Of the studies included, 12 assessed the effect on muscle hypertrophy, 15 on maximal strength, 12 on power, 5 on horizontal displacement, and 7 on vertical displacement. The studies included intervention periods of 4 to 24 weeks with 2–4 sets, 6 to 34 repetitions, and rest periods of 1–5 min between sets. The results from this compilation show that flywheel training appears to be an effective tool to develop strength and induce improvements in functional tests. Below, these effects are discussed in relation to various variables of strength and functional tests.

This meta-analysis only includes studies on healthy individuals. Even though flywheel training was used previously in different types of patient groups for rehabilitation purposes, the results from this review are not necessarily generalizable to patients.

### Strength

All included studies in this meta-analysis showed beneficial results on strength variables after a period of flywheel training.

However, the results, based on effect size, differ for different dependent variables; the beneficial effect on hypertrophy (CSA and muscle volume/mass) had a moderate effect size (0.59 and 0.59), maximal strength showed a very large effect size (1.33), and power showed a large effect size (1.19) from flywheel training (Figs. 4, 5, 6, 7, 8, and 9).

#### Hypertrophy

Twelve of the 20 included studies investigated muscular hypertrophy. It is well known that resistance exercise promotes both neural and muscular adaptations [53–57]. In this meta-analysis, all 11 studies that investigated both muscular hypertrophy and development of maximal strength during the same training period of 5–10 weeks noted a greater relative increase in maximal strength than in hypertrophy. This provides support for previous research suggesting neural adaptations account for the majority of strength development in the initial 3–8 weeks of strength training [1, 54, 56, 58].

The results from this meta-analysis show a mean increase in muscular hypertrophy, muscle volume/mass of 0.20%, and cross-sectional area, 0.19% per day during 5–10 weeks of flywheel training 2–3 times per week. In a comprehensive review by Wernbom et al. [59], they analyzed 44 studies investigating healthy individuals (< 60 years old) and their muscular development of quadriceps femoris and biceps brachii after conventional resistance training with a constant external load. In their review, the authors concluded an increase in muscle hypertrophy of 0.03–0.26% per day and a mean of 0.11% [59]. Interestingly, the average number of days per intervention in the review by Wernbom et al. [59] was 76 days compared to an average of 41 days in the present meta-analysis. The finding of similar total muscular hypertrophy between conventional resistance training and flywheel training, despite substantially shorter study duration in the flywheel studies, is interesting, especially since the muscle hypertrophy response is usually more prominent after several weeks [1, 54, 56, 58].

It is difficult to scientifically compare the difference in training frequency using flywheel training due to the limited number of studies. Earlier data point toward a higher effect after three sessions of conventional resistance training per week compared to once per week, even when training volume was matched [59]. Training frequency was recently suggested to be a key variable to induce muscle hypertrophy [60]. However, there are studies questioning the validity of a larger muscle hypertrophy increase after only 20 days, claiming the initial increase was instead due to edema, as a result of muscle breakdown rather than an actual increase in muscle protein and muscle tissue [61]. If this were to be the case, it could be speculated that the maximal strength would decrease, which was not the case in the studies included in this meta-analysis. In the present data set, there seems to be no correlation between the number of training sessions per week and the increase of muscle CSA. The second greatest muscle hypertrophy response of 0.27% increase per day was noted in a study with only two training sessions per week during a 5-week period [47].

Eight of the 12 included studies measured increases in hypertrophy using MRI, which is considered the gold standard for this type of measurements [62]. In addition, many studies included strategies for minimizing the risk of this error in their study design, for example by allowing the participants to lie down 30–60 min before MRI measurements [63]. Two of the studies performed measurements using DXA, which, according to those authors, correlates well with MRI [27].

For optimal hypertrophy, it is suggested that training should be performed until complete voluntary muscle exhaustion [64]. Significantly higher metabolic and perceptual fatigue was shown with flywheel training compared to resistance training with a Smith machine. [8] An increased physiological stress was also demonstrated, but without significant differences in muscle fatigue when comparing barbell squat training and squats with a flywheel device with the same sets and repetitions [65]. In these studies, the relative load (% of 1RM) was different for the flywheel training (all-out) and the traditional weight training protocols (75–85% of 1RM), potentially affecting the outcome.

#### Maximal Strength

The development of maximal strength was the dependent variable with the largest effect size in this meta-analysis, 1.33. Maximal strength is a combination of both neural and muscular factors. As mentioned previously, the neural factors are responsible for the initial strength increases during the training period. Therefore, the greater relative increase in strength compared to hypertrophy after flywheel training is not surprising [1, 54–56, 58].

It is well documented that untrained individuals experience a greater response in strength than trained individuals [1]. The results from this meta-analysis show, surprisingly, the opposite relationship. The percentage increase in well-trained individuals, 0.41% per day, exceeds the increase in untrained individuals, 0.23% per day. In a meta-analysis on traditional strength training, the authors conclude that untrained individuals develop maximal strength most effectively with moderate loads, corresponding to 60% of 1RM, with four sets, three times per week [66]. Moderately trained individuals, on the other hand, obtain the greatest response with loads at 80% of 1RM, two times per week performing four sets [66]. Well-trained individuals seem to need a greater relative load, 85% of 1RM, and a training volume of eight sets performed two times per week for optimal strength development. However, designing the optimal training program requires consideration of many variables as well as a focus on specificity and individualization. Since study participants included in this meta-analysis were requested to produce maximal acceleration of the flywheel with each repetition, and the number of training sessions per week ranged from 1 to 3 sessions, the resulting load could potentially have become too heavy for the untrained individuals and more optimal for the moderately trained and well-trained participants. One dilemma with such a hypothesis, however, is that higher load requires an increased neural activation which likely is beneficial for strength development [67, 68].

Skeletal muscles have the ability to produce between 20 and 50% more force in the eccentric compared to the concentric phase of the motion [12, 13]. Therefore, maximal eccentric training could be speculated to be an effective way to achieve maximal neural activation.

The ability to activate the muscle seems to differ between trained and untrained individuals [69–71]. The accommodated resistance from the flywheel means that the intensity is completely controlled by the effort of the individual. This allows for a high muscle activation both in the concentric and eccentric phase of the movement, which seems to benefit more trained rather than untrained individuals. A possibility is that stronger individuals, who have some experience in strength training, can be more active during both concentric and eccentric actions, and therefore acquire greater gains. It should also be noted that the mean study period was similar in length for the untrained (44 days) and well-trained group (43 days) in this meta-analysis.

The results from this meta-analysis also show that younger individuals (< 39 years old) [2, 6, 12, 15, 26, 27, 34, 40, 42–44, 47] seem to get greater benefits from flywheel training compared to older individuals (> 59 years old) [36, 41] (0.47% and 0.07% increase per day, respectively). This was observed even though the average number of days per intervention for the older individuals was 63 days compared with 39 days for the younger individuals and with no great difference in training frequency. As we grow older, the amount of muscle mass is reduced, called sarcopenia. This reduction seems to be mediated by both muscular and neural factors, and after 60 years of age, this reduction is accelerated which could be a possible explanation for the higher force development seen in younger individuals compared to older individuals with flywheel training [72, 73].

A relationship exists between the ability of a muscle to develop force and the contraction velocity when lengthened or shortened. As speed in the concentric phase increases, the muscle’s ability to develop high force decreases [74–77]. Therefore, a progressively increased load is recommended for continuous development of strength to avoid the risk of an excessively rapid concentric phase of the movement [11, 62]. None of the included studies used progressively greater inertia during the training period. This means that as the subjects became stronger, the speed of the flywheel increased. Thus, the relative degree of muscle activation probably decreased the stronger the study participants became and the neural activation, therefore, became suboptimal.

As a comparison, a 19% increase in maximal strength was observed after conventional heavy resistance training in untrained middle-aged women and older men during a period of 12 weeks [78]. The results from this meta-analysis show an average increase of 9.6% in maximal strength for untrained individuals. However, this was during a shorter intervention period of 6 weeks, and thus it seems reasonable to conclude flywheel training is as effective as traditional resistance training for the development of maximal strength.

#### Power

Similar to the development of maximal strength, flywheel training seems to cause greater improvements in power for well-trained individuals compared to untrained and moderately trained individuals. The average power development in well-trained individuals was + 0.60% per day, compared to an increase of + 0.44% and + 0.32% for moderately trained and untrained individuals, respectively. Since force together with velocity constitutes power, it is therefore not surprising that power development follows a similar trend as maximal strength following training programs with similar volume and intensity conditions during the intervention period.

### Functional Tests

Tests such as jumps, accelerations, and sprints are classified as functional tests. Improvements in muscle hypertrophy maximal strength and ability to develop power do not always carry over to athletic performance. In contrast, improvements in functional tests usually carry over to a higher extent. Based on the effect sizes, all the investigated components of the functional tests improved; horizontal displacement with a large effect size of 1.01 and vertical displacement also with a large effect size of 0.85. It should be noted that a large effect on functional tests with greater athletic specificity is probably more relevant to athletic performance than a higher effect size in a less functional test (i.e., with less athletic specificity).

#### Horizontal Displacement

This meta-analysis reveals that displacement in the horizontal plane was one of three variables with the highest improvement following flywheel training. The differences in effect size between different distances in short straight sprints could be dependent on the degree of activation of different muscles at different distances. For example, due to changes in running techniques at different velocities, the hamstring musculature, adductor magnus, and gluteus maximus musculature could likely be more activated in 30-m “flying” straight sprints compared to 10-m sprints from a stationary position [79, 80]. The specific muscles used in the training and sprinting would produce greater improvements in one test compared to another, and it is therefore important to choose training and tests based on the principle of specificity.

#### Vertical Displacement

Seven of the 20 included studies measured the effect of flywheel training on functional tests in the vertical direction, e.g., squat jump, countermovement jump, or drop jump. Six of the studies included well-trained individuals and study designs differed regarding the number of sessions per week and the length of the intervention period. The protocol for one study [34] differed from the other studies in that it utilized a high repetition protocol with high velocity. This study showed the greatest improvement, + 10.8%, in vertical displacement, despite having the highest baseline value (44.56 cm) and the shortest intervention period (4 weeks versus 5–24). Thus, a high speed for flywheel training seems effective in improving vertical displacement on functional tests.

## Conclusions

### Practical Application

Flywheel training seems to be a useful load-alternative for development of several variables of strength and to improve results on functional tests. Many of the flywheel machines also offer immediate real-time feedback on several training variables, which can be used to guide training intensity and volume.

Beneficial training protocols with flywheel-based hardware seem to utilize a compressed and intense protocol for a shorter time. Furthermore, according to this meta-analysis, flywheel training appears to be more effective in well-trained individuals than in untrained. Additionally, our analysis showed that younger individuals get a more robust response following flywheel training compared to older individuals.

### Limitations and Future Research Directions

This meta-analysis provides new information on the effectiveness and relevant application of flywheel training for athletes. However, it should be noted that this paper has some limitations. One limitation is the lack of passive control groups in 16 of the 20 included studies. The main reason for using such a design is probably due to the assumption that a control group would likely stay at baseline and flywheel training could, therefore, be compared to a theoretical control group. This supposition is supported by all the studies that used a passive control group [6, 15, 26, 34]. When the number of studies on flywheel training increases and more studies have included a passive control group, including only these studies in a future meta-analysis would provide more robust scientific evidence. Furthermore, one study presenting values for vertical and horizontal displacement was excluded due to lack of complete information [52].

Difficulties arise regarding standardization of range of motion and break force with an accommodated resistance. The accommodated resistance impairs the standardization of methods and the possibility of comparing force production between study participants, thus complicating evaluation of the individual training response.

It should be noted that the degree of inertia has not been considered. This is because inertia is affected by many variables beyond the size, number, and weight of the flywheel, variables that have not been reported in the published studies, e.g., the width of the axis, the thickness of the strap/belt, and the friction coefficient affects the inertia. Thus, the degree of inertia can vary considerably among flywheel machines from different producers despite using the same size, number, and weight of the flywheel. Studies investigating the difference between groups training with different inertia but with a similar number of sets and time under tension fail to show significant differences in maximal strength, power, and functional tests in the vertical direction during an intervention period of 4–5 weeks [6, 26]. An intervention period of 4–5 weeks was probably too short for the inertia to be a crucial factor in these studies.

Finally, the results from this meta-analysis should be interpreted with caution due to the relatively low number of studies included, especially for hypertrophy, horizontal, and vertical displacement. To provide more nuanced analyses and more robust evidence, there is a need for a greater basis of studies investigating and comparing different setups and variables.

At the present date, there is a scarcity of studies investigating relevant outcomes for sports performance, such as change of direction, and level of inertia, from flywheel training. Future research should, therefore, aim to address questions like “Does the effect differ between children and adults?”, “Is flywheel training an effective training modality for improving sports performance in children and adolescents?”, “Can a protocol utilizing a progressively increased resistance (by increasing the inertia as the subjects increase their strength throughout the intervention period) result in superior effects?”

### Summary

Flywheel training is a strength training modality offering the possibility of performing exercises with eccentric overload and variable resistance that therefore differs from conventional gravity-based resistance training. Flywheel training seems to be a viable alternative to regular resistance exercise with comparable positive strength and hypertrophic adaptations in untrained, moderately trained, and well-trained individuals, with, surprisingly, greater strength improvements in the well-trained group and among younger individuals.

In conclusion, flywheel training is an effective method for improving several aspects of strength and power with importance for sports performance.

## Additional File

Additional file 1: **Figure S1.** Funnel plot of studies included for final analysis regarding development of cross sectional area. Figure S2 Funnel plot of studies included for final analysis regarding development of muscle volume/mass. Figure S3 Funnel plot of studies included for final analysis regarding development of maximal strength. Figure S4 Funnel plot of studies included for final analysis regarding development of power. Figure S5 Funnel plot of studies included for final analysis regarding development of functional tests in horizontal direction. Figure S6 Funnel plot of studies included for final analysis regarding development of functional tests in vertical direction. (DOCX 338 kb)

## Abbreviations

1RM: 1 repetition maximum
BIA: Bioelectrical impedance analysis
CI: Confidence interval
CSA: Cross-sectional area
DXA: Dual-energy X-ray absorptiometry
e.g.: Exempli gratia
IV: Inverse variance
MRI: Magnetic resonance imaging
PEDro scale: Physiotherapy Evidence Database scale
PRISMA: Preferred Reporting Items for Systematic Reviews and Meta-analyses
SD: Standard deviation
Std: Standardized

## References

[1] American College of Sports Medicine (2009). American College of Sports Medicine position stand. Progression models in resistance training for healthy adults. Med Sci Sports Exerc.

[2] Norrbrand L, Fluckey JD, Pozzo M, Tesch PA (2008). Resistance training using eccentric overload induces early adaptations in skeletal muscle size. Eur J Appl Physiol..

[3] Roig M, O’Brien K, Kirk G, Murray R, McKinnon P, Shadgan B (2009). The effects of eccentric versus concentric resistance training on muscle strength and mass in healthy adults: a systematic review with meta-analysis. Br J Sports Med..

[4] Ahtiainen JP, Pakarinen A, Alen M, Kraemer WJ, Häkkinen K (2003). Muscle hypertrophy, hormonal adaptations and strength development during strength training in strength-trained and untrained men. Eur J Appl Physiol..

[5] Close RI (1972). Dynamic properties of mammalian skeletal muscles. Physiol Rev..

[6] Naczk M, Brzenczek-Owczarzak W, Arlet J, Naczk A, Adach Z. Training effectiveness of the inertial training and measurement system. J Hum Kinet. 2014;44:19–28. 10.2478/hukin-2014-0107 PMC4327370 25713662

[7] Stone MH, Sanborn K, O’Bryant HS, Hartman M, Stone ME, Proulx C (2003). Maximum strength-power-performance relationships in collegiate throwers. J Strength Cond Res..

[8] Raeder C, Wiewelhove T, Westphal-Martinez MP, Fernandez-Fernandez J, de Paula Simola RA, Kellmann M (2016). Neuromuscular fatigue and physiological responses after five dynamic squat exercise protocols. J Strength Cond Res..

[9] Spiteri T, Newton RU, Binetti M, Hart NH, Sheppard JM, Nimphius S (2015). Mechanical determinants of faster change of direction and agility performance in female basketball athletes. J Strength Cond Res..

[10] de Hoyo M, de la Torre A, Pradas F, Sañudo B, Carrasco L, Mateo-Cortes J (2015). Effects of eccentric overload bout on change of direction and performance in soccer players. Int J Sports Med..

[11] Komi PV (1986). Training of muscle strength and power: interaction of neuromotoric, hypertrophic, and mechanical factors. Int J Sports Med..

[12] Norrbrand L, Pozzo M, Tesch PA (2010). Flywheel resistance training calls for greater eccentric muscle activation than weight training. Eur J Appl Physiol..

[13] Duchateau J, Enoka RM (2016). Neural control of lengthening contractions. J Exp Biol..

[14] Tous-Fajardo J, Gonzalo-Skok O, Arjol-Serrano JL, Tesch P (2016). Enhancing change-of-direction speed in soccer players by functional inertial eccentric overload and vibration training. Int J Sports Physiol Perform..

[15] Seynnes OR, de Boer M, Narici MV (2007). Early skeletal muscle hypertrophy and architectural changes in response to high-intensity resistance training. J Appl Physiol..

[16] Tesch PA, Ekberg A, Lindquist DM, Trieschmann JT (2004). Muscle hypertrophy following 5-week resistance training using a non-gravity-dependent exercise system. Acta Physiol Scand..

[17] Berg HE, Tesch PA (1998). Force and power characteristics of a resistive exercise device for use in space. Acta Astronaut..

[18] Schoenfeld BJ, Ogborn DI, Vigotsky AD, Franchi MV, Krieger JW (2017). Hypertrophic effects of concentric vs. eccentric muscle actions: a systematic review and meta-analysis. J Strength Cond Res..

[19] English KL, Loehr JA, Lee SMC, Smith SM (2014). Early-phase musculoskeletal adaptations to different levels of eccentric resistance after 8 weeks of lower body training. Eur J Appl Physiol..

[20] Friedmann-Bette B, Bauer T, Kinscherf R, Vorwald S, Klute K, Bischoff D (2010). Effects of strength training with eccentric overload on muscle adaptation in male athletes. Eur J Appl Physiol..

[21] Hedayatpour N, Falla D (2015). Physiological and neural adaptations to eccentric exercise: mechanisms and considerations for training. Biomed Res Int..

[22] Maroto-Izquierdo S, García-López D, Fernandez-Gonzalo R, Moreira OC, González-Gallego J, de Paz JA. Skeletal muscle functional and structural adaptations after eccentric overload flywheel resistance training: a systematic review and meta-analysis. J Sci Med Sport. 2017;20:943–51. 10.1016/j.jsams.2017.03.004 28385560

[23] Walker S, Blazevich AJ, Haff GG, Tufano JJ, Newton RU, Häkkinen K (2016). Greater strength gains after training with accentuated eccentric than traditional isoinertial loads in already strength-trained men. Front Physiol..

[24] Douglas J, Pearson S, Ross A, McGuigan M (2017). Chronic adaptations to eccentric training: a systematic review. Sports Med..

[25] Norrbrand L, Tous-Fajardo J, Vargas R, Tesch PA (2011). Quadriceps muscle use in the flywheel and barbell squat. Aviat Space Environ Med..

[26] Naczk M, Naczk A, Brzenczek-Owczarzak W, Arlet J, Adach Z (2016). Impact of inertial training on strength and power performance in young active men. J Strength Cond Res..

[27] Fernandez-Gonzalo R, Lundberg TR, Alvarez-Alvarez L, de Paz JA (2014). Muscle damage responses and adaptations to eccentric-overload resistance exercise in men and women. Eur J Appl Physiol..

[28] Cuenca-Fernández F, López-Contreras G, Arellano R (2015). Effect on swimming start performance of two types of activation protocols. J Strength Cond Res..

[29] de Hoyo M, Pozzo M, Sañudo B, Carrasco L, Gonzalo-Skok O, Domínguez-Cobo S (2015). Effects of a 10-week in-season eccentric-overload training program on muscle-injury prevention and performance in junior elite soccer players. Int J Sports Physiol Perform..

[30] Vicens-Bordas J, Esteve E, Fort-Vanmeerhaeghe A, Bandholm T, Thorborg K (2018). Is inertial flywheel resistance training superior to gravity-dependent resistance training in improving muscle strength? A systematic review with meta-analyses. J Sci Med Sport..

[31] Nuñez Sanches FJ, Saez de Villarreal E. Does flywheel paradigm training improve muscle volume and force? A meta-analysis. J Strength Cond Res. 2017:3177–86. 10.1519/JSC.0000000000002095. 10.1519/JSC.0000000000002095 29068866

[32] Downs SH, Black N (1998). The feasibility of creating a checklist for the assessment of the methodological quality both of randomised and non-randomised studies of health care interventions. J Epidemiol Community Health..

[33] Liberati A, Altman DG, Tetzlaff J, Mulrow C, Gøtzsche PC, Ioannidis JPA (2009). The PRISMA statement for reporting systematic reviews and meta-analyses of studies that evaluate healthcare interventions: explanation and elaboration. BMJ..

[34] Naczk M, Naczk A, Brzenczek-Owczarzak W, Arlet J, Adach Z (2016). Efficacy of inertial training in elbow joint muscles: influence of different movement velocities. J Sports Med Phys Fitness..

[35] Cronin JB, Hing RD, McNair PJ (2004). Reliability and validity of a linear position transducer for measuring jump performance. J Strength Cond Res..

[36] Bruseghini P, Calabria E, Tam E, Milanese C, Oliboni E, Pezzato A (2015). Effects of eight weeks of aerobic interval training and of isoinertial resistance training on risk factors of cardiometabolic diseases and exercise capacity in healthy elderly subjects. Oncotarget..

[37] Gual G, Fort-Vanmeerhaeghe A, Romero-Rodríguez D, Tesch PA (2016). Effects of in-season inertial resistance training with eccentric overload in a sports population at risk for patellar tendinopathy. J Strength Cond Res..

[38] Glatthorn JF, Gouge S, Nussbaumer S, Stauffacher S, Impellizzeri FM, Maffiuletti NA (2011). Validity and reliability of Optojump photoelectric cells for estimating vertical jump height. J Strength Cond Res..

[39] Pueo B, Lipinska P, Jiménez-Olmedo JM, Zmijewski P, Hopkins WG (2017). Accuracy of jump-mat systems for measuring jump height. Int J Sports Physiol Perform..

[40] Askling C, Karlsson J, Thorstensson A (2003). Hamstring injury occurrence in elite soccer players after preseason strength training with eccentric overload. Scand J Med Sci Sports..

[41] Caruso JF, Hamill JL, Hernandez DA, Yamauchi M (2005). A comparison of isoload and isoinertial leg press training on bone and muscle outcomes. J Strength Cond Res..

[42] Lundberg TR, Fernandez-Gonzalo R, Gustafsson T, Tesch PA (2012). Aerobic exercise does not compromise muscle hypertrophy response to short-term resistance training. J Appl Physiol..

[43] Lundberg TR, Fernandez-Gonzalo R, Tesch PA (2014). Exercise-induced AMPK activation does not interfere with muscle hypertrophy in response to resistance training in men. J Appl Physiol..

[44] Maroto-Izquierdo S, García-López D, de Paz JA (2017). Functional and muscle-size effects of flywheel resistance training with eccentric-overload in professional handball players. J Hum Kinet..

[45] Núñez FJ, Santalla A, Carrasquila I, Asian JA, Reina JI, Suarez-Arrones LJ (2018). The effects of unilateral and bilateral eccentric overload training on hypertrophy, muscle power and COD performance, and its determinants. in team sport players. PLoS One..

[46] Onambélé GL, Maganaris CN, Mian OS, Tam E, Rejc E, McEwan IM (2008). Neuromuscular and balance responses to flywheel inertial versus weight training in older persons. J Biomech..

[47] Owerkowicz T, Cotter JA, Haddad F, Yu AM, Camilon ML, Hoang TN (2016). Exercise responses to gravity-independent flywheel aerobic and resistance training. Aerosp Med Hum Perform..

[48] Sabido R, Hernández-Davó JL, Botella J, Navarro A, Tous-Fajardo J (2017). Effects of adding a weekly eccentric-overload training session on strength and athletic performance in team-handball players. EJSS..

[49] Walker E, Hernandez AV, Kattan MW (2008). Meta-analysis: Its strengths and limitations. Cleve Clin J Med..

[50] Cohen J (1988). Statistical power analysis for the behavioral sciences.

[51] Sawilowsky SS (2009). New effect size rules of thumb. J Mod Appl Stat Methods..

[52] de Hoyo M, Sañudo B, Carrasco L, Domínguez-Cobo S, Mateo-Cortes J, Cadenas-Sánchez MM (2015). Effects of traditional versus horizontal inertial flywheel power training on common sport-related tasks. J Hum Kinet..

[53] Kraemer WJ, Adams K, Cafarelli E, Dudley GA, Dooly C, Feigenbaum MS, et al. American College of Sports Medicine position stand. Progression models in resistance training for healthy adults. Med Sci Sports Exerc. 2002;34(2):364–80. 10.1097/00005768-200202000-00027 11828249

[54] Higbie EJ, Cureton KJ, Warren GL, Prior BM (1996). Effects of concentric and eccentric training on muscle strength, cross-sectional area, and neural activation. J Appl Physiol..

[55] Moritani T, deVries HA (1979). Neural factors versus hypertrophy in the time course of muscle strength gain. Am J Phys Med..

[56] Narici MV, Roi GS, Landoni L, Minetti AE, Cerretelli P (1989). Changes in force, cross-sectional area and neural activation during strength training and detraining of the human quadriceps. Eur J Appl Physiol Occup Physiol..

[57] Aagaard P, Simonsen EB, Andersen JL, Magnusson P, Dyhre-Poulsen P (2002). Neural adaptation to resistance training: changes in evoked V-wave and H-reflex responses. J Appl Physiol..

[58] Gabriel DA, Kamen G, Frost G (2006). Neural adaptations to resistive exercise: mechanisms and recommendations for training practices. Sports Med..

[59] Wernbom M, Augustsson J, Thomeé R (2007). The influence of frequency, intensity, volume and mode of strength training on whole muscle cross-sectional area in humans. Sports Med..

[60] Morton RW, McGlory C, Phillips SM (2015). Nutritional interventions to augment resistance training-induced skeletal muscle hypertrophy. Front Physiol..

[61] Damas F, Phillips SM, Lixandrão ME, Vechin FC, Libardi CA, Roschel H (2016). Early resistance training-induced increases in muscle cross-sectional area are concomitant with edema-induced muscle swelling. Eur J Appl Physiol..

[62] Reeves ND, Maganaris CN, Narici MV (2004). Ultrasonographic assessment of human skeletal muscle size. Eur J Appl Physiol..

[63] Berg HE, Tedner B, Tesch PA (1993). Changes in lower limb muscle cross-sectional area and tissue fluid volume after transition from standing to supine. Acta Physiol Scand..

[64] Goto K, Ishii N, Kizuka T, Takamatsu K (2005). The impact of metabolic stress on hormonal responses and muscular adaptations. Med Sci Sports Exerc..

[65] Timón R, Ponce-González JG, González-Montesinos JL, Olcina G, Pérez-Pérez A, Castro-Piñero J. Inertial flywheel resistance training and muscle oxygen saturation. J Sports Med Phys Fitness. [Epub ahead of print July 24, 2017; Available from 10.23736/S0022-4707.17.07793-3]. 10.23736/S0022-4707.17.07793-3 28738671

[66] Peterson MD, Rhea MR, Alvar BA (2005). Applications of the dose-response for muscular strength development: a review of meta-analytic efficacy and reliability for designing training prescription. J Strength Cond Res..

[67] Kawamori N, Haff GG (2004). The optimal training load for the development of muscular power. J Strength Cond Res..

[68] Schoenfeld BJ, Peterson MD, Ogborn D, Contreras B, Sonmez GT (2015). Effects of low- vs. high-load resistance training on muscle strength and hypertrophy in well-trained men. J Strength Cond Res..

[69] Seger JY, Thorstensson A (2000). Electrically evoked eccentric and concentric torque-velocity relationships in human knee extensor muscles. Acta Physiol Scand..

[70] Duchateau J, Semmler JG, Enoka RM (2006). Training adaptations in the behavior of human motor units. J Appl Physiol..

[71] Douglas J, Pearson S, Ross A, McGuigan M (2017). Eccentric exercise: physiological characteristics and acute responses. Sports Med..

[72] Jespersen J, Pedersen TG, Beyer N (2003). Sarcopenia and strength training. Age-related changes: effect of strength training. Ugeskr Laeger..

[73] Marzetti E, Leeuwenburgh C (2006). Skeletal muscle apoptosis, sarcopenia and frailty at old age. Exp Gerontol..

[74] Sale DG, MacDougall JD, Alway SE, Sutton JR (1987). Voluntary strength and muscle characteristics in untrained men and women and male bodybuilders. J Appl Physiol..

[75] Wickiewicz TL, Roy RR, Powell PL, Perrine JJ, Edgerton VR (1984). Muscle architecture and force-velocity relationships in humans. J Appl Physiol..

[76] Westing SH, Seger JY, Thorstensson A (1990). Effects of electrical stimulation on eccentric and concentric torque-velocity relationships during knee extension in man. Acta Physiol Scand..

[77] Hortobágyi T, Katch FI (1990). Role of concentric force in limiting improvement in muscular strength. J Appl Physiol..

[78] Häkkinen K, Kallinen M, Linnamo V, Pastinen UM, Newton RU, Kraemer WJ (1996). Neuromuscular adaptations during bilateral versus unilateral strength training in middle-aged and elderly men and women. Acta Physiol Scand..

[79] Delecluse C (1997). Influence of strength training on sprint running performance. Current findings and implications for training. Sports Med..

[80] Brughelli M, Cronin J, Chaouachi A (2011). Effects of running velocity on running kinetics and kinematics. J Strength Cond Res..

