# Supplementary material for: Effects of Flywheel Training on Strength-Related Variables: a Meta-analysis
Source: Sports Med Open. 2018 Dec 13;4:55. doi: 10.1186/s40798-018-0169-5 (PMC6292829; doi:10.1186/s40798-018-0169-5)
Supplement: Supplementary file 1 — Figure S1. Funnel plot of studies included for final analysis regarding development of cross sectional area. Figure S2 Funnel plot of studies included for final analysis regarding development of muscle volume/mass. Figure S3 Funnel plot of studies included for final analysis regarding development of maximal strength. Figure S4 Funnel plot of studies included for final analysis regarding development of power. Figure S5 Funnel plot of studies included for final analysis regarding development of functional tests in horizontal direction. Figure S6 Funnel plot of studies included for final analysis regarding development of functional tests in vertical direction. (DOCX 338 kb) [file 40798_2018_169_MOESM1_ESM.docx]

**Additional file 1**

#### **Funnel plots**

**
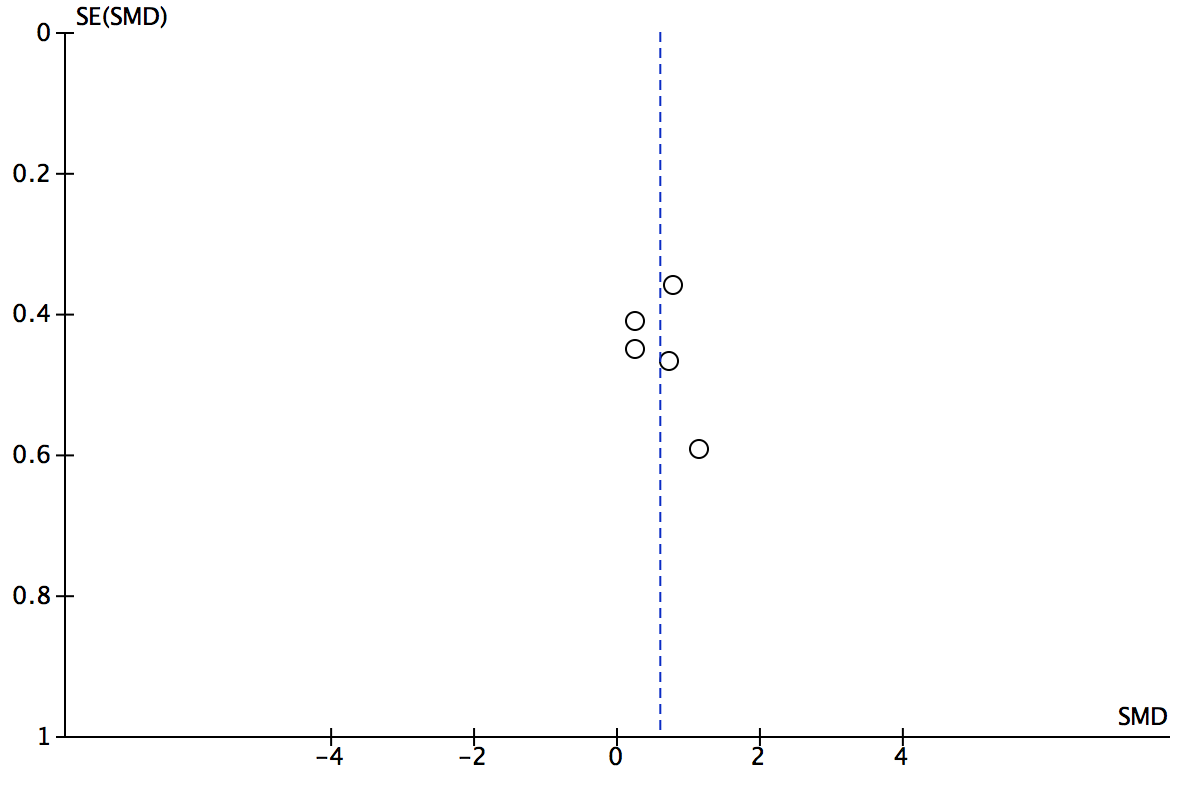
**

**Additional file 1: Figure S1.** Funnel plot of studies included for final analysis regarding development of cross sectional area.


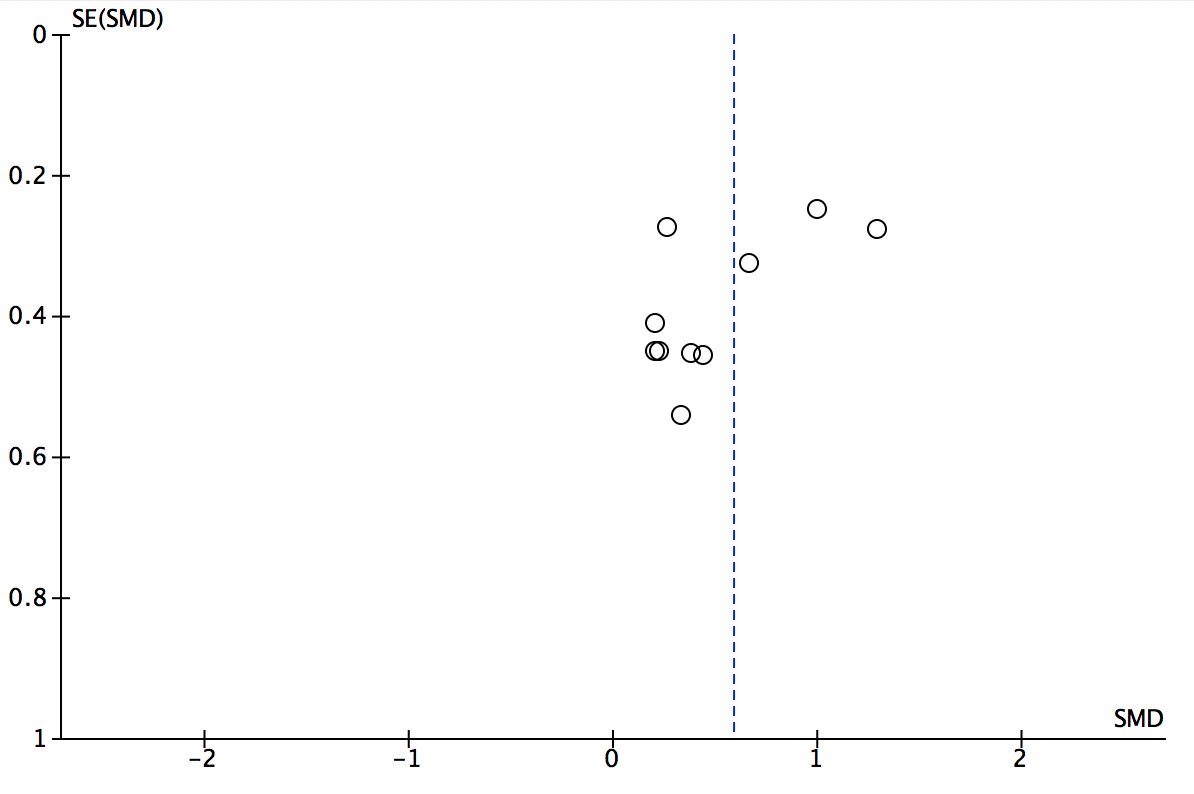


**Additional file 1: Figure S2.** Funnel plot of studies included for final analysis regarding development of muscle volume/mass.


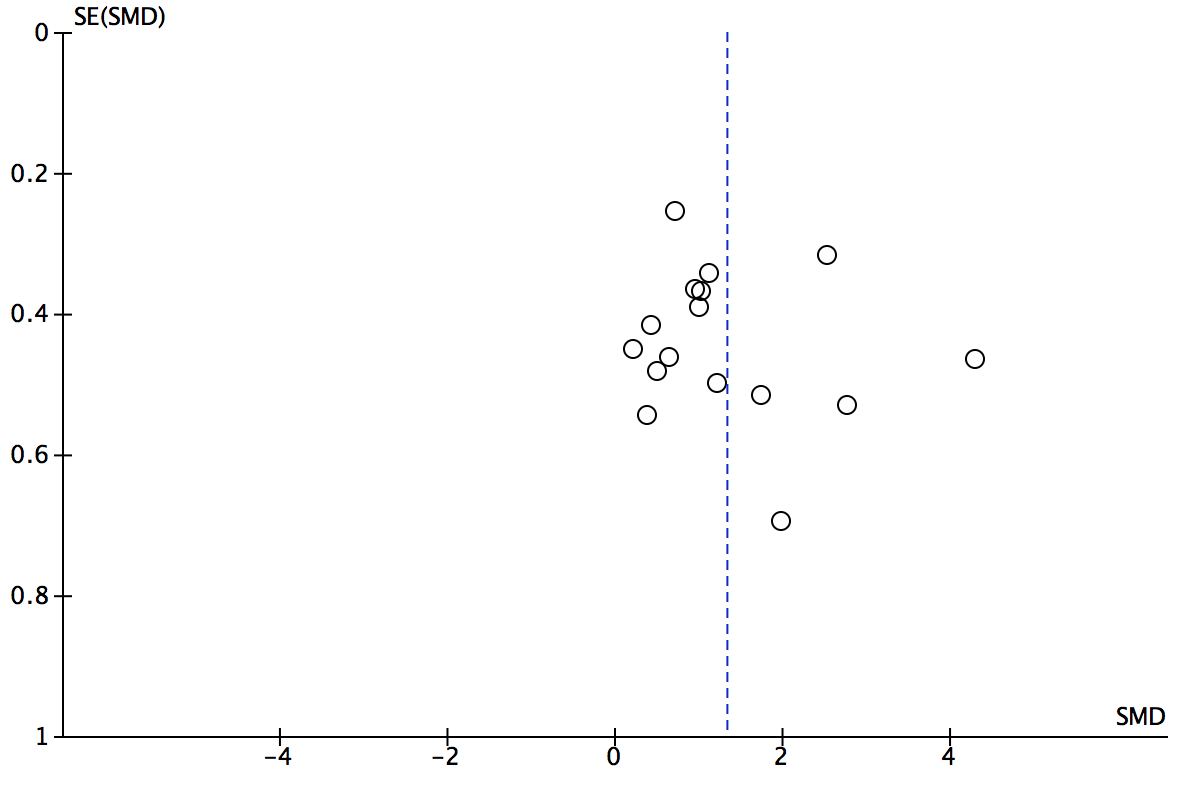


**Additional file 1: Figure S3.** Funnel plot of studies included for final analysis regarding development of maximal strength.


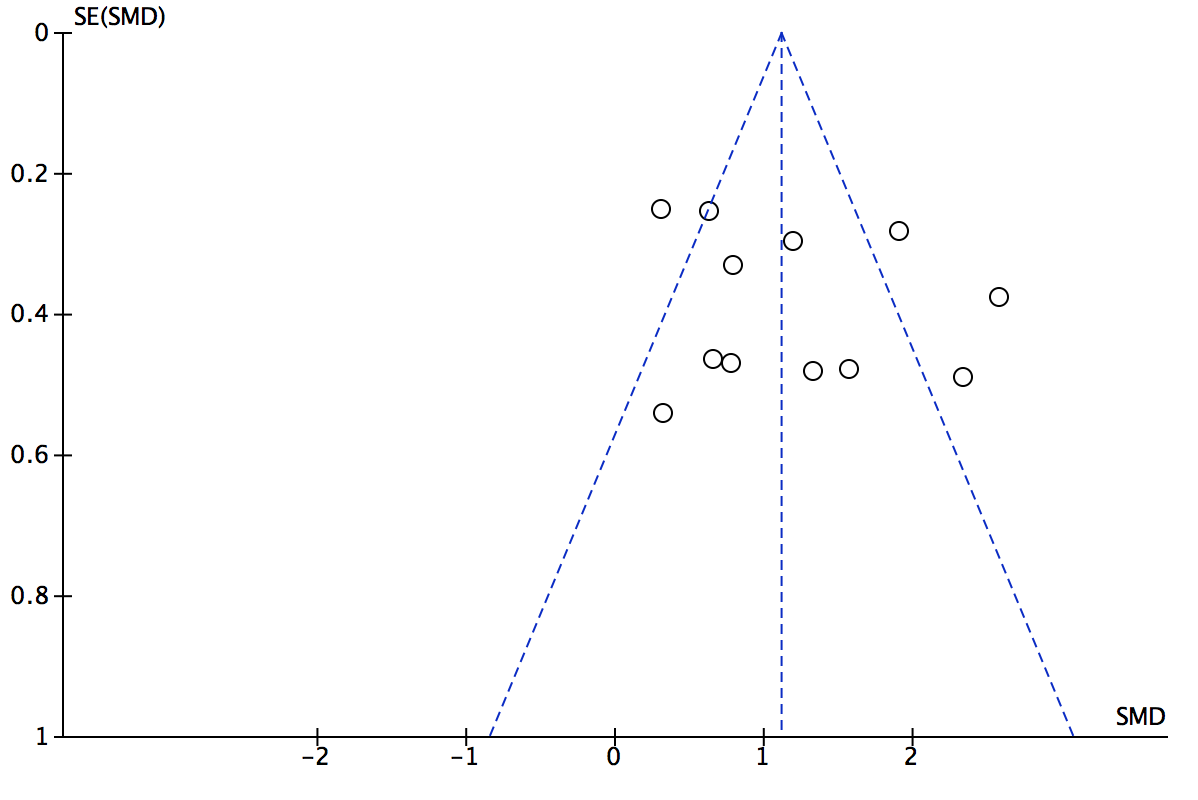


**Additional file 1: Figure S4.** Funnel plot of studies included for final analysis regarding development of power.


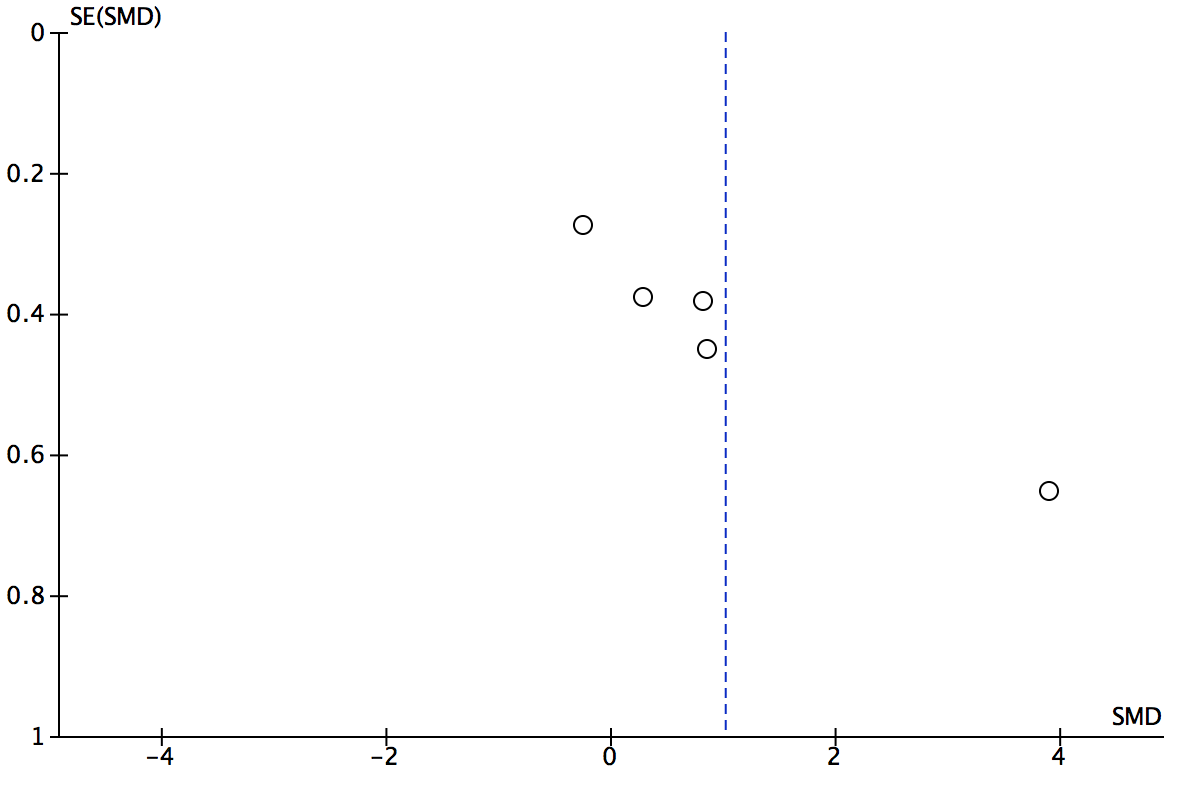


**Additional file 1: Figure S5.** Funnel plot of studies included for final analysis regarding development of functional tests in horizontal direction.


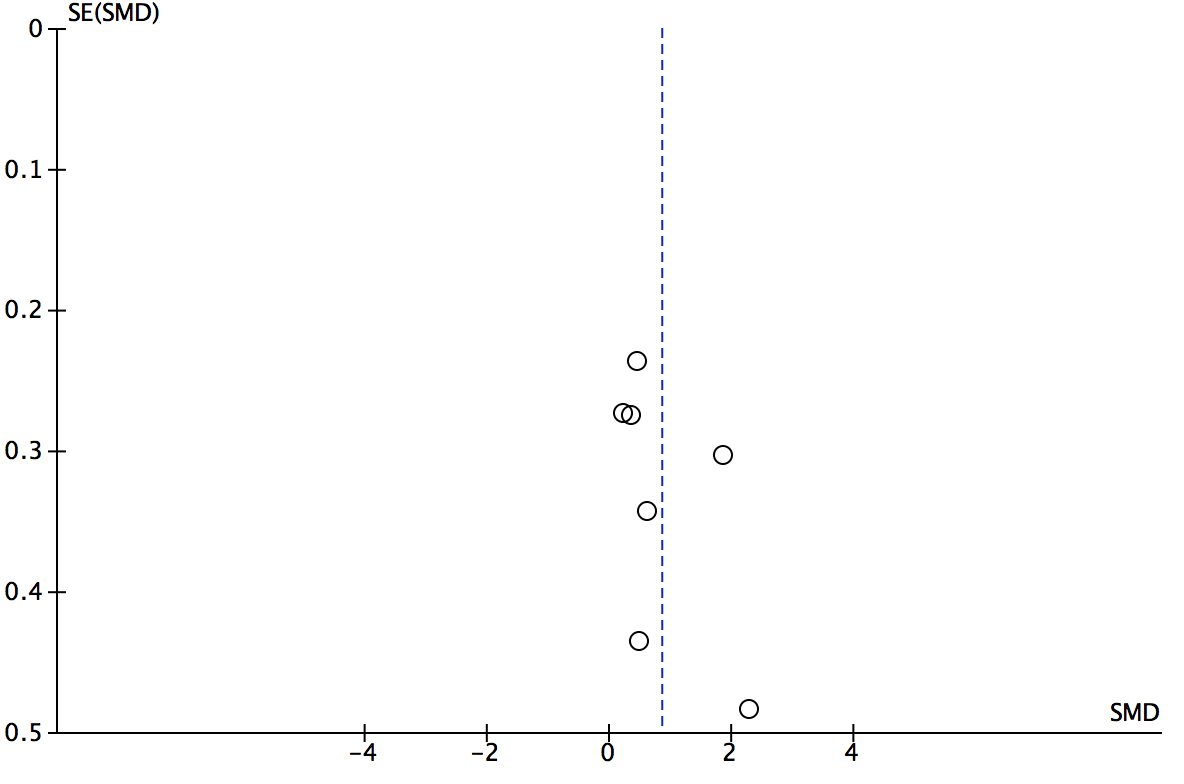


**Additional file 1: Figure S6.** Funnel plot of studies included for final analysis regarding development of functional tests in vertical direction.
